# Application of Optical Laser 3D Surface imaging system (Sentinel) in breast cancer radiotherapy

**DOI:** 10.1038/s41598-020-64496-1

**Published:** 2020-05-05

**Authors:** Mengjiao Liu, Xiaobo Wei, Yun Ding, Changhai Cheng, Wenming Yin, Jie Chen, Kou Wang, Wendong Gu

**Affiliations:** Department of Radiation Oncology, The Third Affiliated Hospital of Soochow University, 185 Juqian Street, Changzhou, 213003 People’s Republic of China

**Keywords:** Breast cancer, Cancer therapy

## Abstract

It has been clearly confirmed that radiation therapy (RT) after breast conserving surgery (BCS) is an effective treatment modality comparable to mastectomy for early breast cancer. The purpose of this study was to further evaluate the accuracy of 3D surface imaging system (Sentinel) for breast cancer patients received BCS. The optical surface scans and CBCT scans were acquired before and immediately after couch movement correction. The deviation of the CBCT scans from the reference planning CT was considered an estimate for the residual errors for patient setup correction. The planning target volume (PTV) margins for treatment sessions was calculated according to the setup errors. We obtained a total of 245 sets of data collected from 49 breast cancer patients. Compared with Sentinel setup errors, the residual setup errors as determined by the CBCT scans after couch movement correction were reduced in the six directions. The PTV margins derived from the CBCT residual errors were all less than 5 mm in X, Y, and Z directions. Our results suggested that Optical surface imaging can be applied in positioning for breast cancer patient accurately without unnecessary imaging dose.

## Introduction

Breast cancer is the most common cancer with the highest morbidity in females worldwide, with more than 1.2 million cases diagnosed every year, accounting for 10–12% of the global female population and killing an average of 500,000 people every year. The 10-year overall survival for patients who received adjuvant radiotherapy versus BCS alone increased by 12%^1^. Postoperative combined radiotherapy not only significantly improved the local tumor control rate, but also achieved better long-term survival rate than the operation alone^2^. A randomized controlled trial including the Cancer Institute of Milan and NSABPB 06 showed no significant difference in tumor-free survival between breast-conserving surgery plus breast radiotherapy and mastectomy.

Conventional radiotherapy involves two tangential field irradiations to minimize lung and heart exposure. However, the different distance between different parts of the mammary gland and the special shape of the mammary gland often cause the dose uneven in the target area. For 3D conformal and intensity modulated radiation therapy techniques (IMRT) promise the radiation dose homogeneity within the planning target volume, at the same time reduce the radiation dose delivered to the contralateral breast, which may reduce toxicity and improve local control^3^. However, accurate patient positioning is crucial for the use of highly conformal radiotherapy techniques. Image-guided radiation therapy (IGRT) can improve the accuracy of positioning by reducing the distance between the clinical target volume (CTV) and the planned target volume (PTV)^4^. However, this technique uses ionizing radiation which increase the extra imaging dose to patients^5^. Moreover, the flow of CBCT in assisting setup generally takes a long time^6^. The new scanning method of optical surface imaging was equipped with accurate and no additional ionizing radiation in assisting setup. Previously, we have collected 27 cases of breast cancer patients who underwent radiation therapy after breast-conserving surgery, and analyzed the setup accuracy of optical surface imaging by the Sentinel system and its correlation with CBCT. The results were optimistic. In this study, we collected 49 breast cancer patients with the same conditions to further study the accuracy of 3D surface imaging system (Sentinel).

## Results

We obtained a total of 245 sets of data collected from 49 breast cancer patients. The numbers of treatment sessions for individual patient were from 4 to 7, and the median was 5.

The variation analysis of SE and CBCT residual errors were given in Table 1. The CBCT residual errors after correction of the patient’s position by use of Sentinel were all smaller than SE in three directions, and the *p*-values were less than 0.001.

**Table 1:** The variation analysis of Sentinel errors and CBCT residual errors.

	SE	CBCT residual errors	P-value
X(left-right)	−1.45 mm ± 4.44 mm	0.94 mm ± 2.65 mm	<0.001
Y(cranial-caudal)	−1.97 mm ± 4.72 mm	1.64 mm ± 3.11 mm	<0.001
Z(anteroposterior)	−2.62 mm ± 3.98 mm	1.21 mm ± 2.57 mm	<0.001
Rx(x-rotation)	0.5° ± 0.9°	0.3° ± 0.9°	<0.001
Ry(y-rotation)	0.2° ± 0.6°	0.3° ± 1.0°	<0.001
Rz(z-rotation)	0.0° ± 0.5°	0.0° ± 0.8°	<0.001

SE: sentinel errors; CBCT: cone-beam computerized tomography.

The Σ and δ of residual errors based on the CBCT scanned after the couch movement according to SE were shown in Table 2, these errors were comparable between the two alignment methods with the differences in six directions by 0.97 mm and 0.80 mm, 0.72 mm and 0.60 mm, 1.03 mm and 0.38 mm, 0.1°and 0.2°, 0.4°and 0.1°, 0.3°and 0.2°, respectively. It can be seen that after correction of the patient’s positions by Sentinel, CBCT residual errors were all reduced in six directions. The PTV margins derived from the data were reduced to 4.40 mm, 4.99 mm, 4.62 mm in X, Y, and Z directions, respectively.

**Table 2:** Setup errors of Sentinel and residual setup errors of CBCT.

		The group mean (M)	Systemic errors (∑)	Random errors (σ)	PTV margin
X-shift (mm)	CBCT (mm)	0.71	1.73	1.34	4.40
	Sentinel (mm)	−1.83	2.70	2.14	6.89
Y-shift (mm)	CBCT (mm)	1.62	1.90	1.70	4.99
	Sentinel (mm)	−2.00	2.62	2.30	6.85
Z-shift (mm)	CBCT (mm)	0.84	1.60	2.03	4.62
	Sentinel (mm)	−3.00	2.63	2.41	6.95
X-rotation (°)	CBCT (°)	0.5	0.8	0.6	—
	Sentinel (°)	0.3	0.9	0.8	—
Y-rotation (°)	CBCT (°)	0.2	0.6	0.8	—
	Sentinel (°)	0.2	1.0	0.9	—
Z-rotation (°)	CBCT (°)	0.0	0.5	0.7	—
	Sentinel (°)	0.0	0.8	0.9	—

PTV: planning target volume.

## Discussion

It has been clearly confirmed that radiation therapy (RT) after breast conserving surgery (BCS) is a treatment modality comparable to mastectomy for early breast cancer^7^. Increasing the dose of target area radiotherapy is the key to improving the local control rate of breast cancer. Since the spatial position of breast and surrounding normal tissues is constantly changing during the treatment, if failure to pay sufficient attention to these changes and errors may result in the increase of tumor miss and normal tissue damage, thus reducing the curative effect. One of the influencing factors of the location uncertainty in the radiotherapy process is the random error in the position of the irradiation field: refers to the position difference caused by the technician’s positioning state during each treatment and the breast position changes during the treatment. Clinical practice and experimental studies have confirmed that the errors will have a significant impact on the dose distribution of tumor target area and surrounding normal tissues, especially in conformal and intensity-modulated radiotherapy.

IGRT has substantially increased total score accuracy in radiation therapy by minimizing setup errors and organ motion uncertainties^8^, electronic field imaging system (EPID), CBCT and other devices have been able to conduct more accurate research on the uncertainty of target area, including verification of position and dose, and correction by offline and online methods. The new EPID and CBCT is installed on the accelerator to perform location verification as well as dose distribution calculation and verification. However, X-ray based IGRT process involves additional radiation to patients^9^. According to the study, the dose in the field of single CBCT scan was about 1.5 ~ 3.0 cGy, and the highest dose was up to 7.2cGy^10^. The additional dose of CBCT scan not only increases the risk effect of tissue in the irradiated area, but also increases the risk of random carcinogenic effect. Followill^11^ *et al*. found that IMRT can significantly increase the incidence of the second primary tumor. They estimated the incidence of a second primary tumor at a dose of 70 Gy: 0.3 percent when two-dimensional irradiation, and a significant increase (up to 1 percent) when IMRT due to a sharp increase in the accelerator machine unit (MU).

In addition, the flow of CBCT acquisition, reconstruction, and registration may takes 2–5 min, which prolongs treatment time at each fraction, where a 15–20 min time slot is typically used^6^. Therefore, a technique that can improve the accuracy of radiotherapy without additional radiation is needed clinically, namely, surface imaging techniques.

Pallotta *et al*.^12^ compared the difference between Sentinel and CBCT measurement errors by using the medical up to six dimensional bed and Alderson Rando human phantom, the results showed that the absolute values of the difference between Sentinel and CBCT measured and known errors in the six directions of shift and rotation were all less than 0.5 mm and 0.3°. Deantonio^13^ *et al*. studied the application of AlignRT system to position verification in three-dimensional conformal radiotherapy for breast cancer. They compared the difference of positioning errors (up and down and forward and backward) between AlignRT system and EPID verification. The results show that although the AlignRT system may be affected by respiratory movements, its translational error is less than 0.5 mm compared with EPID, so it is considered that the AlignRT system can partially replace EPID in the auxiliary placement of three-dimensional conformal radiotherapy for breast cancer. Gierga *et al*.^14^ found that the average differences between the kV-based clip registration and the surface imaging were 1.3 mm, 1.4 mm, 1.8 mm in X, Y, Z directions respectively for breast cancer. Alderliesten *et al*.^15^ studied the setup errors of surface imaging system for monitoring intrafraction motion in stereotactic body radiotherapy of lung cancer. They suggested that surface imaging system can monitor intrafraction motion errors in SBRT for female lung cancer patients. It can be seen from many research results that the differences between surface imaging system and CBCT are small. Surface imaging technology has certain value in radiotherapy positioning.

In our previous study, we found that optical surface imaging by Sentinel has a significant correlation with CBCT in detecting setup errors in postoperative radiotherapy for breast cancer. The differences of Σ and δ between the two methods were less than 1 mm. In addition, we detected the residual setup errors based on the Sentinel surface imaging scanned after the couch movement. The PTV margins derived from the data were all decreased by 1~2 mm in all directions^16^. All of these results show that Sentinel system has high accuracy for assisting patient setup. In this study, we further verify the setup accuracy of Sentinel in assisting setup for breast cancer patients. The results showed that the CBCT residual errors after correction of the patient’s position by use of Sentinel were all smaller than SE in X, Y, and Z directions. In addition, the Σ and δ of residual errors based on the CBCT scanned after the couch movement according to SE were all reduced in six directions. The PTV margins derived from the residual errors were less than 5 mm in X, Y, and Z directions.

Holmes *et al*.^17^ found after verification of CBCT guidance setup based on rib or surface registration, the residual setup errors for breast tumor bed boost averaged 3.0 mm. Batin *et al*.^18^ studied the residual errors of AlignRT assisted setup for breast cancer after modified radical mastectomy and found that the residual setup errors were 2.9 ± 1.5 mm, 1.4 ± 1.4 mm and 2.2 ± 1.4 mm in three translational directions, respectively. Moreover, a study demonstrated that PTV margin were required to expand 3.5 mm, 2.4 mm, 4.0 mm in three directions according to the residual errors detected by Optical surface imaging system in breast cancer^19^. These results are in agreement with our study.

A number of studies have confirmed the accuracy of positioning by surface imaging system. The study performed by Schoeffel *et al*.^20^ showed a high stability and accuracy of surface imaging system for positioning of breast cancer patients, The setup accuracy in vertical axis was always less than 0.3 degrees. G Li *et al*.^21^ found that the motion detection accuracy was less than 0.1 mm and 1 degree by using surface‐image‐guided head repositioning in six directions. Alderliesten *et al*.^22^ studied the correlation of AlignRT™ system and CBCT setup errors of breast cancer patients after BCS. They found that the correlation coefficients between AlignRT™ system with CBCT in three translational directions were 0.70, 0.90 and 0.82, respectively, which were all significantly correlated.

Although our study has achieved optimistic results, Optical Laser 3D Surface imaging system is currently rare in China, and this is a single-center study. Therefore, it may be difficult to gather more patients. This directly leads to the small sample size of this study, which lacks certain persuasion. In future clinical work, we will continue adding additional cases to expand the study sample size. Furthermore, the Sentinel system was self-deviated due to the high relative position mobility of the abdominal and pelvic tumors, which would cause changes in the body contour of the patients, so we only studied setup errors in radiotherapy in breast cancer patients after breast conserving surgery. To make this study more definitive, we will continue to collect patients with head and neck tumors undergoing radiotherapy to further verify the accuracy of the Sentinel system.

## Materials and Methods

The same parts of the methods were cited from our previous research^16^.

### General clinical information

This was a prospective study had approved from the Medical Ethics Committee of the Third Affiliated Hospital of Soochow University, and written informed consent was obtained from the patients before treatment. The methods used in this study were carried out in accordance with the guidelines outlined in the Declaration of Helsinki. Our study included 49 patients who received radiotherapy after breast cancer conserving surgery. The median age of the patients was 52 years old (range 41–64 years). The case number for intraductal carcinoma and invasive carcinoma of the study population were 10 and 39, the case numbers for T_is_, T_1_, and T_2_ stage were 10, 22, and 17, respectively; the N and M stage for all cases were N_0_ and M_0_. The characteristics of the study population are shown in Table 3.

**Table 3:** Characteristics of the study population (n = 49).

Clinical characteristics	Value
**The pathologic types**	
Intraductal carcinoma (n)	10
Invasive carcinoma (n)	39
**The pathological staging**	
pTisN0M0 (n)	10
pT1N0M0 (n)	22
pT2N0M0 (n)	17
Mammary gland volume(cm^3^)	467.9 ± 173.4
Age (years old)	52.5 ± 7.0
The average BMI (kg/m^2^)	24.3 ± 2.9

BMI: body mass index.

### Patient positioning procedure and treatment planning

Patients were immobilized using a breast board (Posirest-2 Arm Support, CIVCO, US) in supine position with arms raised above the head^16^. Planning CT scan was performed on a Sensation Open CT scanner (Siemens, Germany). CT images with a slice thickness of 5 mm covered the region from the annular membrane to the lower boarder of liver. Three longitudinal laser lines and one transversal laser line were marked on patient’s skin surface, and their intersecting points were tattoo marked for setup positioning at treatment.

For treatment planning, the CTV was delineated to include all breast tissues. The PTV was defined as the CTV with a 5 mm margin, modified to exclude the volume adjacent to the skin surface within 5 mm. The CTV-boost volume consisted of the tumor bed, as marked by the surgery metal clips, postoperative serum swelling and surgical scars. The PTV-boost was defined as adding 7 mm margin to the CTV-boost and modified to be inside the PTV. Organ as risk (OAR) delineated were the lung, heart, and contralateral breast.

Dose prescription was 57.5 Gy in 25 fractions for PTV-boost and 47.5 Gy in 25 fractions for PTV. The dosimetric objectives were to cover 95% of the target volumes with the prescription dose and limit the OAR dose such that the V5 of ipsilateral lung was less than 50%, the mean dose to the heart less than 6 Gy, and the maximum dose of contralateral breast less than 5 Gy.

Treatment plans were generated using the Monaco treatment planning system (TPS) (Monaco 3.3, Elekta, Sweden). A single 190° arc was used for the beam setup. A 6MV photon beam was used. For the left-side treatment the arc started at gantry angle of 300° and ended at 130°, and for the right-side treatment the gantry angles were from 60° to 230°. The maximum number of control points was 150.

### Sentinel scan and CBCT scan

**Figure 1:**
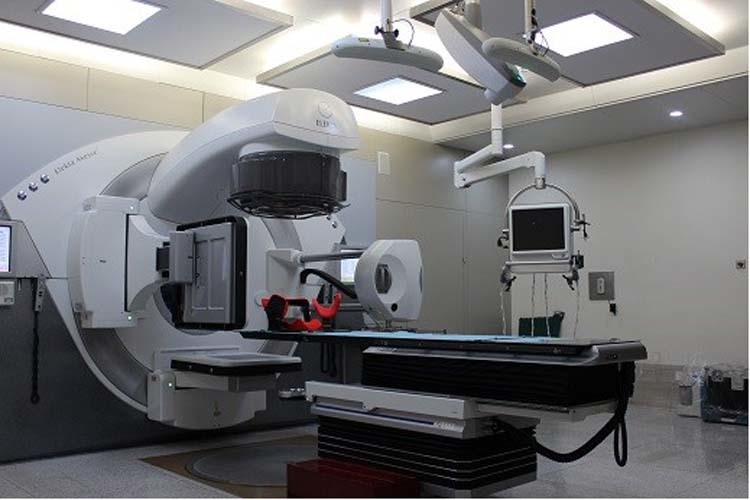
The image of Sentinel and CBCT positioning correction equipment.

**Figure 2:**
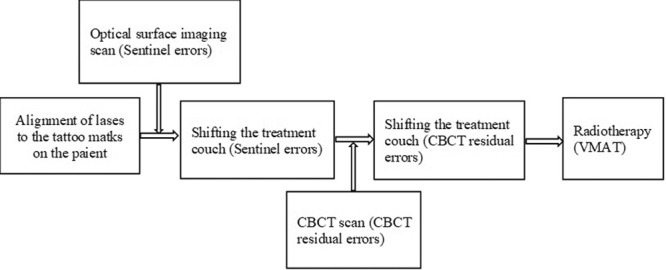
Flow chart of research steps.

This study was designed for further evaluating the patient setup accuracy of using the optical surface imaging in the clinical setting but without deviating from the current CBCT based method for patient positioning correction. The starting point of patient setup was the alignment of treatment room lasers to the tattoo marks on the patient. Patient skin surface was acquired using the Sentinel system (C-RAD, Sweden), and the region of interest (ROI) consisted of the disease side breast and the adjacent chest wall. The upper boundary was the clavicle, and the medial boundary was the midline. The contralateral breast, chin, arm and armpit were excluded. The matching of the acquired surface ROI with the surface reconstructed by the planning CT yielded setup corrections that can be applied by a 6-dimensional (6D) treatment couch (HexaPOD, Elekta, Sweden). The shifts in the X, Y, Z directions and the three rotations about the X, Y, Z axes were recorded as a data set named SE (Sentinel errors before couch movement), and the correction was applied using the automatic couch movement function for the HexaPOD couch. Next, the CBCT scan was performed, and the setup errors as determined by the C-RAD software was denoted as CBCT residual setup errors. Prior to treatment delivery, the second correction was applied using the automatic couch movement function for the HexaPOD couch. The modified procedure to conduct the current study was facilitated by the MOSAIQ (Elekta, Sweden) Oncology Information System. In this workflow, it was the attending physician, Mengjiao Liu and Wendong Gu, and the technician, Xiaobo Wei, analysed the images and data for the first time, and then finished by the same technician, Xiaobo Wei. The image of Sentinel and CBCT positioning correction equipment is shown in Fig. 1, and a step by step procedure is shown in Fig. 2.

### Statistical method

Data were analyzed using the SPSS 21.0 software and were represented as mean ± standard deviation ($\bar{x}$ ± SEM)^16^. All results were obtained from three independent replicates. The group mean (M) of the study cohort was the average of the averaged error for each patient. The systematic error (Σ) was the standard deviation (SD) of the individual mean for each patient, and the randomize error (δ) was the root mean square of the mean square deviation of each patient^23^, The PTV margins were calculated by 2Σ + 0.7δ, based on the work by Stroom *et al*.^24^.

## Conclusion

Optical surface imaging by Sentinel system is a fast and non-invasive method for assisting patient setup. Our study showed that Sentinel system could help in the positioning of breast cancer patients. However, 3D surface systems have the limitation of a superficial acquisition which does not provide internal information. We suggested that Sentinel system can be applied in positioning for tumors with small organ movements such as the chest and head and neck.
